# Acute Sleep Deprivation and Circadian Misalignment Associated with Transition onto the First Night of Work Impairs Visual Selective Attention

**DOI:** 10.1371/journal.pone.0001233

**Published:** 2007-11-28

**Authors:** Nayantara Santhi, Todd S. Horowitz, Jeanne F. Duffy, Charles A. Czeisler

**Affiliations:** 1 Division of Sleep Medicine, Department of Medicine, Brigham & Women's Hospital, Harvard Medical School, Boston, Massachusetts, United States of America; 2 Division of Sleep Medicine, Harvard Medical School, Boston, Massachusetts, United States of America; 3 Visual Attention Laboratory, Brigham & Women's Hospital, Cambridge, Massachusetts, United States of America; 4 Department of Ophthalmology, Harvard Medical School, Boston, Massachusetts, United States of America; University of St. Andrews, United Kingdom

## Abstract

**Background:**

Overnight operations pose a challenge because our circadian biology promotes sleepiness and dissipates wakefulness at night. Since the circadian effect on cognitive functions magnifies with increasing sleep pressure, cognitive deficits associated with night work are likely to be most acute with extended wakefulness, such as during the transition from a day shift to night shift.

**Methodology/Principal Findings:**

To test this hypothesis we measured selective attention (with visual search), vigilance (with Psychomotor Vigilance Task [PVT]) and alertness (with a visual analog scale) in a shift work simulation protocol, which included four day shifts followed by three night shifts. There was a nocturnal decline in cognitive processes, some of which were most pronounced on the first night shift. The nighttime decrease in visual search sensitivity was most pronounced on the first night compared with subsequent nights (p = .04), and this was accompanied by a trend towards selective attention becoming ‘fast and sloppy’. The nighttime increase in attentional lapses on the PVT was significantly greater on the first night compared to subsequent nights (p<.05) indicating an impaired ability to sustain focus. The nighttime decrease in subjective alertness was also greatest on the first night compared with subsequent nights (p<.05).

**Conclusions/Significance:**

These nocturnal deficits in attention and alertness offer some insight into why occupational errors, accidents, and injuries are pronounced during night work compared to day work. Examination of the nighttime vulnerabilities underlying the deployment of attention can be informative for the design of optimal work schedules and the implementation of effective countermeasures for performance deficits during night work.

## Introduction

The ubiquitous ‘night shift’ of modern 24-hour society is a challenge to our biological propensity for daytime wakefulness and nighttime sleep. The ensuing misalignment between the circadian timing system and the sleep-wake schedule during night work impairs many waking functions [1], [2]. Because the deterioration in cognitive functioning at the circadian nadir worsens as sleep pressure increases [3], [4], individuals are most vulnerable to its consequences after extended wakefulness [5]. The *first night in a sequence of night shifts*, typically preceded by ∼16 hours of wakefulness provides such a situation [6]. Unfortunately this window of vulnerability has been somewhat neglected in research and by policy makers, even though 3.2% (∼864000) of full time wage and salary earners in the United States work the night shift [7] with a significant number of those earners presumably in transition from a day to night shift at any given time.

While a prophylactic nap can ameliorate effects of extended wakefulness [8], in the real world many shift workers are either unable or choose not to nap. Moreover, operational needs sometimes require workers to be scheduled for a ‘quick comeback’ where they work the day followed by a night shift with only an 8 hour break in between, leaving little opportunity for sleep. In such circumstances, workers could be awake for 24 consecutive hours by the end of the first night shift [6], a duration of wakefulness associated with increased risk of errors, accidents and injuries [5] comparable to those associated with alcohol intoxication [9] [10]–[12].

We investigated nighttime impairment in attention, a diverse psychological phenomenon [13]–[16] which includes selective attention (the ability to process relevant information to the exclusion of irrelevant information [17], [18]) and vigilance (the ability to sustain focus for an extended period of time [19], [20]). Nighttime deficits in selective attention [15] are less well understood than nighttime deficits in vigilance [13], [21]–[24] although both could affect safety and productivity in the workplace. In fact, our ability to ignore irrelevant or distracting items is vital for effective performance in many critical round the clock operations such as baggage screening at airports, power plant maintenance and air traffic control. In this study, we measured selective attention with two visual search tasks [18], [25], assessed vigilant attention with the Psychomotor Vigilance Task (PVT) [26](detailed in methods) and subjective alertness with a visual analog scale.

**Figure 1:**
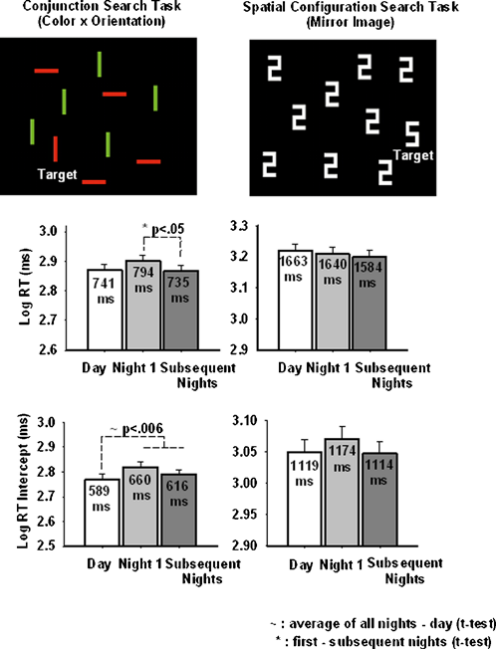
Stimuli and Response Time Data from the Visual Search Tasks. The top panels show a representative target present trial in the two search tasks. The top left panel shows a conjunction search trial where the target is a red vertical bar and the distractors, green vertical and red horizontal bars. The top right panel shows a spatial configuration search trial where the target a white block numeral ‘5’, and the distractors, the mirror image white block numeral ‘2s’. The middle and lower panels show the log transformed RT and the RT x set size intercept results. In the middle and lower panels, the x-axis represents the work shift. In the middle panels the y-axis represents RT and in the bottom panels it represents the RT intercept, both in log units. The error bars represent the standard error of the mean. The day shift (baseline) data are shown in the white bar and represent the average of the third and fourth day shifts (the first two shifts were excluded to minimize practice effects; see text for details); night 1 data are shown in the light gray bar; data from the ‘subsequent’ nights are shown in the dark gray bar, and represent the average of the second and third night shifts. The middle panels show RT data from the two search tasks. As seen in the left panel, RT was slowest on the night 1 in the conjunction search task, while the right panel indicates that this slowing did not occur in the spatial configuration search task. The lower panels show RT x set size intercept data from the two search tasks. As seen in the left panel RT intercept during night work was significantly slower than during day work in the conjunction search task (t-tests).

Visual search requires subjects to find a target item in a display cluttered with distractor items. By varying the total number of items presented (set size), we can decompose response time (RT) into two separate components reflecting attentional and non-attentional stages of processing (see ***Selective Attention*** below) [18], [27]. While previous sleep deprivation protocols have employed other variants of the visual search paradigm, they did not vary set size, making it difficult to separate effects on selective attention per se from general response slowing [28] [29]–[31]. One study which did vary set size reported a type of speed-error trade-off in visual search: sleepy subjects tended to engage in faster but less accurate search [15]. To understand the implications of this impairment in the context of shift work, we included a similar visual search paradigm in our protocol. Deficits in attentional tasks can occur in the early preattentive stage and/or the later selective attentional stage of the task [18]. Because the difficulty level of the visual search task influences the relative contribution of these two stages of processing, we employed two different visual search tasks, an easy conjunction search task (Figure 1, left panel) and a more difficult spatial configuration search task (Figure 1, right panel) in order to better determine where deficits in processing occur.

The PVT requires subjects to monitor a source of information over time, for the occurrence of an infrequent, unpredictable stimulus [26], [32]. Changes in the PVT RT of sleepy subjects are considered to reflect *lapsing*, a heightened tendency for microsleep which can lead to missed stimuli or extremely slow responses, and *cognitive slowing*, the overall increase in response latencies [33]. The visual analog scale required subjects to rate their level of alertness on a 100 mm line labeled at end with the endpoints of the alertness dimension labeled as ‘sleepy’ and ‘alert’.

Our protocol included four day shifts (07:00 to 15:00) followed by three night shifts (23:00 to 07:00)[34]. After the final day shift, the subjects were scheduled to sleep that night at their usual time, had a free day, and then began the first night shift 32 hours after the end of the last day shift. On the day shifts subjects slept at night. During the night shift part of the study, each subject was randomly assigned to one of two sleep schedules, either 08:00-16:00 or 14:00 to 22:00 [34]. We found that the nighttime loss of alertness was accompanied by a slowing of response and decline in accuracy due to loss of sensitivity and increased lapsing, both of which were most acute on the first night shift.

## Results

### Statistical Analysis

We analyzed our data using the statistical package SAS (version 9.1) and SPSS (Graduate student version 12.0) for personal computers. The visual search and PVT data were analyzed using a mixed model ANOVA, which is effective in controlling for individual variability. Random effects were specified with an intercept-slope model and the residual maximum likelihood method was used for fitting the model. The variance components formed the covariance structure and the containment method was used to compute the degrees of freedom. Backward elimination was used to systematically reduce the ANOVA terms (α = .05 significance level). Relevant main effects and interaction effects were further examined using t-tests. Because there was no effect of sleep schedules on our cognitive measures we pooled the data from both sleep schedule groups for the analyses reported here. The analyses of RT (measured in milliseconds [ms]) were all done on the log transformed data.

ANOVA Factors: For all tasks, work episode (*baseline* (average of the third and fourth day shifts), *first night shift*, and ‘*subsequent nights’* (average of second and third shifts) and session (2 (beginning of a shift), 3 (middle of a shift) and 4 (end of a shift)) were the within-subjects factors. The visual search tasks included additional within-subjects factors. For the RT analyses the additional within-subjects' factors were target (present vs. absent) and set size (10, 20, 30, or 40). In the visual search tasks, set size was omitted as a factor in the analysis of the RT slope and RT intercept because they are computed as a function of set size. Target was omitted as a factor in the d′ (“d prime”, the sensitivity parameter) analysis because it is computed using the hit rate and false alarm rate.

Planned Comparisons: (1) to determine nighttime deficit, the overall nighttime performance was compared to daytime (baseline) performance, (2) to determine whether deficits were most pronounced on the first night shift, performance on the first night shift was compared to ‘subsequent nights’ performance, and (3) to determine the decline over the course of a shift, performance in session 4 was compared to performance in session 2 within the same shift.

Due to time constraints, the conjunction search task was not presented during the first session of each work shift. To maintain consistency in our analyses between performance tests, we did not use data from the first testing session in any of our analyses. The visual search data included 3 sessions administered every two hours (conjunction search: starting at 9:45 a.m. on day shifts and 1:45 a.m. on night shifts; spatial configuration search starting at 9:30 a.m. on day shifts and 1:30 a.m. on night shifts). The PVT data included 3 sessions administered every two hours starting at 10:30 a.m. on day shifts and at 2:30 a.m. on night shifts. Data from the first two day shifts were excluded in order to minimize practice effects on the tasks (analyses of the slope of the RT x set size function and accuracy in visual search indicated an improvement over the first two day shifts, but not from day 3 to day 4). Data from the second and third night shifts were combined since our initial analyses revealed that there were no significant differences in performance between those two nights.

### Selective Attention

In a search paradigm, the mean RT is an overall measure of speed of processing. However, the actual search time (an index of selective attention) can be separated from sensory, post-search decision and response times by manipulating the set size (number of stimuli in the array) and computing the linear RT x set size function [35]. Search time is reflected in the slope of this function, while the other components of RT are relegated to the intercept [18]. In addition to speed, the paradigm also provides accuracy information. We analyzed d′ computed from z-transformed hit and false alarm rates, which has the advantages of being independent of response bias and being normally distributed [36]. In the ANOVA model, target and set size were tested as fixed effects and subject as a random effect. Work episode and session were tested as both fixed and random effects, the latter in order to account for individual intercept and slope differences in these factors.

### The effect of target presence and set size effects on visual search performance

Based on the visual search literature [18], we expected to find effects of target (target present RTs tend to be faster than target absent RTs), set size (increase in RT with set size) and the interaction between target and set size (the effect of set size would be larger on target absent trials) on RT, in our ANOVA. First, these analyses indicated a main effect of target on the mean RT, RT intercept and RT slope (Table 1; p<.001). In both search tasks, target present trials were faster than target absent trials and target present slopes were shallower than target absent slopes. Second, the ANOVA revealed a main effect of set size on RT (Table 2; p<.001) in both search tasks, in that there was a significant increase in mean RT with set size. Finally, our data suggested that the change in RT with set size was bigger on the target absent trials and this was confirmed by the significant target x set size interaction on RT in our ANOVA (Table 2; p<.001). These effects which reflect underlying properties of visual search are typical for search tasks with high target prevalence.

**Table 1:** Effect of Target on Response Time Measures in Visual Search

Task	Independent variable	Log Transformed RT	Log Transformed RT Intercept	Log Transformed RT Slope
*Conjunction Search*				
	*Target Present*	**2.82±.02** [*661 ms*]	**2.77±.02** [589 ms]	**.002±.0004** [*3 ms/item*]
	*Target Absent*	**2.94±.02** [*871 ms*]	**2.82±.02** [661 ms]	**.0048±.0004** [*12 ms/item*]
	*Target Effect*	F_(1,2003)_ = 1180.86; p<.001	F_(1,329)_ = 31.88; p<.001	F_(1,17)_ = 19.30; p<.001
*Spatial Configuration Search*				
	*Target Present*	**3.07±.03** [1175 ms]	**2.94±.02** [871 ms]	**.005±.0004** [15 ms/item]
	*Target Absent*	**3.35±.03** [2239 ms]	**3.12±.02** [1318 ms]	**.007±.0004** [41 ms/item]
	*Target Effect*	F_(1,1571)_ = 3805.32; p<.001	F_(1,329)_ = 910.52; p<.001	F_(1,329)_ = 59.76; p<.001

This table represents the main effect of target from the ANOVA—a standard effect in visual search where target present trials have a faster response time (RT) than target absent trials. The log transformed response time (RT), RT intercept, and RT slope in both the conjunction search task and in the spatial configuration search task show this effect.

**Table 2:** Effect of Set Size on Response Time and Sensitivity (d′) in Visual Search

Task	Independent Variable	Log Transformed RT	d'
*Conjunction Search*	*10*	**2.83±.02** [*676 ms*]	**3.32±.03**
	*20*	**2.87±.02** [*741 ms*]	**3.36±.03**
	*30*	**2.90±.02** [*794 ms*]	**3.31±.03**
	*40*	**2.92±.02** [*832 ms*]	**3.29±.03**
	*Set Size Effect*	F_(3,2003)_ = 165.72; p<.001	F_(3.51)_ = 1.39; p>.05
	*Target x Set Size Interaction*	F_(3,2003)_ = 33.5; p<.001	
*Spatial Configuration Search*			
	*10*	**3.09±.03** [*1230 ms*]	**3.32±.03**
	*20*	**3.20±.03** [*1584 ms*]	**3.16±.03**
	*30*	**3.26±.03** [*1820 ms*]	**3.12±.03**
	*40*	**3.29±.03** [*1950 ms*]	**2.93±.04**
	*Set Size Effect*	F_(3,1571)_ = 399.55; p<.001	F_(3,51)_ = 17.01; p<.001
	*Target x Set Size Interaction*	F_(3,1571)_ = 9.91; p<.001	

This table represents the main effect of set size from the ANOVA—a standard effect in visual search representing an increase in RT and a decrease in d′ with set size, which is an index of search difficulty. The effect of set size on RT was greater with the target absent than with the target present trials in both search tasks as indicated by the target x set size interaction. There was an effect of set size on d′ in the spatial configuration search task, but not in the conjunction search task, reflecting that the conjunction search task is an easier search than the spatial configuration search.

### Longer mean RT and RT intercept during night shifts indicate slower responding

To test our hypothesis of nighttime impairment we looked at the effects of work episode and session on the mean RT and RT x set size intercept, from the ANOVA. Changes in response speed during the different work shifts were primarily seen in conjunction search, where we found a significant effect of work episode on the mean RT (F_2, 34_  = 3.4; p = .05). Planned comparisons (t-tests) indicated that responses were slowest on the first night shift (59 ms slower than the subsequent nights; Figure 1, middle left panel). The ANOVA also indicated a significant work episode x session interaction on RT (F_4, 2003_ = 10.57; p<.001), where nighttime responses were 55 ms slower at the end of shift relative to the beginning, while daytime responses were 25 ms faster by the end of the shift. Moreover, the difference between the target present and target absent trials was greatest on the first night shift (first night shift: 232 ms vs.; ‘subsequent nights’: 166 ms) as indicated by a significant the work episode x target interaction on RT (F_2, 2003_ = 8.19; p<.001).

Like mean RT, nighttime changes in the RT intercept were primarily seen in conjunction search (Figure 1). The RT intercept is thought to reflect sensory, perceptual, decision and response stages of processing and is less weighted by the selective attention stage. Our ANOVA revealed a significant effect of work episode on the RT intercept in the conjunction search task (F_2, 34_ = 3.87; p = .03). Planned comparisons (t-tests) indicated that the average nighttime RT intercept was significantly longer (by 49 ms) than the daytime RT intercept (Figure 1, bottom left panel). In the spatial configuration search task, although the increase in RT intercept seemed to be most pronounced on the first night shift, it did not reach statistical significance (Figure 1, bottom right panel). There were no other significant effects or interactions in this analysis.

### Shallower RT slope during night shifts indicate faster search rate, a selective attentional deficit

**Figure 2:**
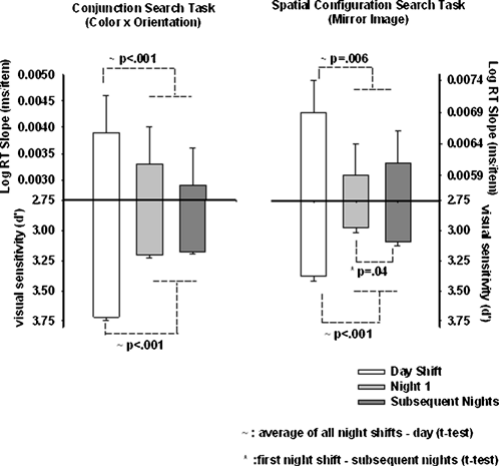
Speed/Accuracy Trade Off in Selective Attention. The data in this figure represent the slopes of the RT x set size function (index of selective attention) in log units and d' (an index of sensitivity reflecting accuracy) in the visual search tasks. The error bars represent the standard error of the mean. As in figure 1 the x-axis represents the work shift. In the upper part of the figure the y-axis represents RT slope in (msecs per additional item). In both search tasks, the decreasing values of RT slope during night work suggest that there was either a speed-up in search with increasing number of items or there was a failure in processing information arising from nocturnal cognitive impairment. In the lower part of the figure the y-axis represents d'. Decreasing values of d' during night work in both search tasks indicate a loss of accuracy. In the spatial configuration search task (right panel) this loss of accuracy was greatest on the first night shift. Together the results presented in this figure suggest a speed-error trade-off on the night shifts, indicating that participants either failed to collect sufficient information or there was a failure in processing information arising from nocturnal cognitive impairment.

To test our hypothesis of night shift impairment we looked at the effect of work episode and session on the RT slope, from the ANOVA. The RT slope is considered an index of selective attention because it is thought to reflect the cost of attending to each additional item [18], [27]. Shallower RT slopes indicate faster search [18], [27]. The ANOVA showed a significant effect of work episode in both the conjunction search (F_2,34_ = 8.31; p = .001) and spatial configuration search tasks (F_2, 34_ = 3.64; p = .04). Interestingly, planned comparisons (t-tests) indicated that the slopes were actually significantly shallower during night work relative to day work, in both search tasks (Figure 2, upper panels). This suggests that as set size increased subjects spent less time attending to each item. There were no other significant main effects or interactions between any of the factors.

### Lower d′ during night shifts reflects loss of accuracy

As with RT measures, changes in accuracy with set size reflect changes in search behavior, while changes in the overall level of accuracy reflect more central or peripheral processes. We combined hit and false alarm rates to compute d′, the standard signal detection measure of sensitivity, also an index of accuracy. Lower d′ values indicate decreased sensitivity to the presence of the target. Typically, d′ ranges from 0 (chance performance) to 4.0. Our ANOVA revealed a main effect of set size on d' in the spatial configuration task (F_2, 34_ = 17.01; p<.001) in that d′ decreased with set size (Table 2, column 4). This effect was absent in the conjunction search task, reflecting that conjunction search is easier than spatial configuration search. There were no interactions between set size and the other factors in the analysis.

To test our hypothesis of night shift impairment we looked at the effect of work episode on the d′ from the ANOVA. This analysis indicated a significant effect of the work episode on d′ in both the conjunction (F_2, 760_ = 171.47; p<.001) and spatial configuration (F_2, 34_ = 51.52; p<.001) search tasks. Planned comparisons (t-tests) showed that there was a significant nighttime decrement in d' in both search tasks (Figure 2, lower panels). More notably, this decline in sensitivity (confirmed by a t-test) was most pronounced on the first night shift in the more difficult spatial configuration search task (Figure 2, lower right panel). There were no other significant main effects or interactions between the factors.

In summary, our visual search data indicated that subjects searched faster, spent less time per item searching, yet responded more slowly and less accurately on the night shifts. In the more difficult spatial configuration search the decline in accuracy as reflected by a loss of sensitivity was most pronounced on the first night shift.

### Vigilance

**Figure 3:**
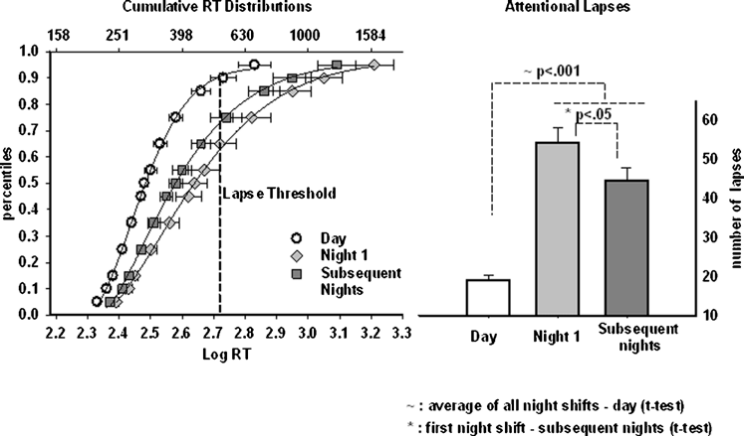
The Impact of Night Work on Vigilance RT and Attentional Lapses. This figure presents results from the PVT task. The left panel represents the group-averaged cumulative response time (RT) percentile distribution. The x-axis represents response time with the bottom x-axis in log units and the top x-axis in milliseconds. The error bars represent the standard error of the mean. The dashed vertical line is plotted at the average attentional lapse threshold (90th percentile of baseline RT). The y-axis represents percentile points. The average cumulative distributions were computed by calculating the RT percentiles for the day (open circles), first night shift (filled diamond) and ‘subsequent’ night shifts (filled square) for each individual subject, and then averaging them across subjects to compute the final cumulative distributions. For each of the shifts, these cumulative distributions were fitted with a 4-parameter Weibull function. The right panel presents the number of attentional lapses on the three shifts. The error bars represent the standard error of the mean. There was a significant increase in lapses during night work, and this was most pronounced on the first night shift.

To test our hypothesis of nighttime impairment in vigilance we focused on the effects of work episode and session on two aspects of the RT distribution: the cumulative RT percentile distribution [37]–[39]and lapses. To calculate the average cumulative distribution we first computed the 5^th^, 10^th^, 15^th^, 25^th^, 35^th^, 45^th^, 50^th^, 55^th^, 65^th^, 75^th^, 85^th^, 90^th^ and 95^th^ percentiles for the day, first night and subsequent night shifts individually for subject. These individual percentile values within each shift category were then averaged across subjects to compute the final cumulative distributions for the day, first night and subsequent night shifts (Figure 3, left panel). A 4-parameter Weibull function provided the best fit description for each cumulative distribution. In the ANOVA model subject was tested as a random effect. Work episode and session were tested as both fixed and random effects.

### Longer RT during night shift suggests cognitive slowing

While we cannot decompose RTs from the PVT into different processing components [40] the way we can with visual search RTs, we can analyze two independent aspects of the RT distributions. The extent of cognitive slowing, the increase in the time needed to execute a simple response can be measured by changes in the standard quartiles (median, 25^th^, and 75^th^ percentiles). Meanwhile, outliers in the RT distribution are thought to reflect lapses, when attention is not focused on the task.

The ANOVA indicated a significant effect of work episode on the median RT (F_2, 34_ = 23.6; p<0.001), 25^th^ percentile RT (F_2, 34_ = 40.07; p<.001) and 75^th^ percentile RT (F_2, 34_ = 11.73; p<.001). Planned comparisons (t-tests) confirmed that there was a significant nighttime increase (see relevant data points in Figure 3, left panel) in all three measures (the median RT, p<.001; 25^th^ percentile RT, p<.001; and 75^th^ percentile RT, p = .001). Note that even the fastest RTs (i.e. the 25^th ^percentile) were slower at night than during the day. Furthermore, planned comparisons (t-tests) also revealed that the slowest RT's (Figure 3, left panel) were significantly longer on the first night shift compared to the subsequent nights (p = .02). There were no other significant effects or interactions.

### Attentional lapses were most pronounced on the first night shift

We defined the 90^th^ percentile of the daytime baseline as the threshold for an attentional lapse (average lapse threshold shown in Figure 3, left panel) and determined this individually for each subject. The number of RTs exceeding this threshold for the day, first night and subsequent nights was then computed as attentional lapses for each subject. The data shown in the right panel of Figure 3 are the lapses for each shift averaged across subjects.

To test our hypothesis of the night shift impairment we looked at the effect of work episode and session on lapses from our ANOVA. This analysis indicated a significant effect of the work episode (F_2, 34_ = 27.05; p<0.001); planned comparisons (t-tests) indicated that there was a nighttime increase in lapses (Figure 3, right panel), and that this was most pronounced on the first night shift (Figure 3, right panel). In the ANOVA, we also found a significant effect of session (F_2, 34_ = 4.46; p = .02) and a marginally significant work episode x session interaction (F_4,68_ = 2.23; p = .08) on the lapses. A closer examination of the data revealed that there was a 28% increase in lapses by the end of the shift compared to the beginning of the shift during night work but not during day work.

In summary, the PVT data indicated that subjects responded more slowly and had more attentional lapses during the night shifts than during day shifts and that lapses were most pronounced on the first night shift.

### Subjective Alertness

The visual analog scale of subjective alertness ranged from 1 to 100, where higher values represented higher levels of alertness. The effects of work episode and session were most relevant for our hypothesis of nighttime impairment.

### Nighttime decrement in subjective alertness was most pronounced on the first night shift

The ANOVA revealed a significant effect of the work episode on subjective alertness (F_2,101_ = 67.8; p<0.001); planned comparisons (t-tests) indicated that alertness levels were lower during night work relative to day work (day: 71.3+3.8 vs. night: 52.3+3.2; p<.001) and that it was lowest on the first night shift (night 1: 39±4.05 vs. subsequent nights: 52.3±4.05; p<.05). Furthermore, the ANOVA also showed a significant effect of session (F_2, 34_ = 3.43; p = .04) and a significant work episode x session interaction (F_4, 101_ = 3.01; p = .02). Planned comparisons (t-tests) revealed that subjective alertness declined significantly by the end of the shift during night work (p<.01) but not during day work.

## Discussion

We found a nighttime loss of alertness accompanied by a slowing of response, a decline in accuracy due to loss of sensitivity and increased lapsing [41] [42] [43] [10]. These nocturnal deficits provide some insight into why occupational errors, accidents and injuries are highly prevalent in overnight operations. Our most significant findings pertain to nighttime impairment in selective attention and to those nocturnal deficits in attention and alertness that were most pronounced on the first night shift. We found that on the night shifts, subjects responded more slowly and less accurately than they did on day shifts even though they searched faster—they spent less time attending to each item. More significantly, the nocturnal decline in search accuracy (d′) was most acute on the first night shift, albeit only in the more challenging task. To our knowledge this is the first detailed examination of the impact of night work on selective attention using the visual search paradigm. The acute loss of accuracy (d′) on the first night shift was accompanied by a pronounced increase in attentional lapses and a decline in alertness, which points to the detrimental impact the first night of work can have on safety and productivity in the workplace.

### Speed and accuracy of selective attention during night work

Although loss of accuracy on night shifts has been widely reported in the literature, due to the nature of the tasks used, it is normally difficult to trace the source of errors from these data [5], [24], [41], [44]. Such information would not only aid our understanding of the cognitive underpinnings of performance in a task, but it could be invaluable for ergonomically optimizing task environments to reduce errors in the workplace. Loss of accuracy in tasks involving complex visual information can result from either a decline in sensitivity or a change in response bias [36]; with a decline in d′ we can infer a loss of sensitivity. Sensitivity in visual search tasks not only degrades substantially during night work (Figure 2 lower panels), but it appears to be amplified on the first night shift when the search task becomes more challenging. This acute deterioration in sensitivity points to an interaction between the combined effects of sleep loss/circadian misalignment and task difficulty. From a theoretical standpoint this argues strongly for including the role of the circadian system and sleep homeostat as factors when modeling selective attention. From a practical standpoint this has implications for optimizing nighttime working environment in jobs that involve complex visual tasks, for instance by increasing the signal to noise ratio in the visual displays to enhance sensitivity.

Perhaps the most intriguing result from our visual search tasks was that the nighttime decrease in accuracy as a function of set size was accompanied by a trend towards shallower RT slopes (Figure 2 upper panels), similar to what Horowitz et al [15] found in a sleep deprivation study. The fact that in both studies subjects failed to compensate for this loss of accuracy by increasing search time appropriately as the number of items increased, even with feedback (speed and accuracy) on every trial, implies impaired decision making. This decision making impairment persisted through all the night shifts. It is also interesting to note that in the conjunction search task faster search rates (shallower slopes) were accompanied by longer RT's showing that subjects were not *responding* too quickly, they were *searching* too quickly.

A comparison of performance in the two search tasks is informative as well. The effects of work episode in the RT domain were stronger in conjunction search relative to spatial configuration search. The opposite effect was observed in spatial configuration search. Because conjunction search is substantially more efficient (by a factor of three or four in this study), perceptual and motor components are more heavily weighted in RT analysis of this search relative to that of spatial configuration search. In the spatial configuration search task, because the selective attention component is more heavily weighted, RT effects are smaller. At the same time, spatial configuration search is more difficult, so the effects of the speed-error trade-off are magnified in this task. Thus, more difficult search tasks may be more sensitive for detecting selective attention impairments. By the same token jobs involving difficult search tasks, e.g. radiology, some military operations may be more vulnerable to loss of performance efficiency arising from nocturnal selective attention deficits.

### Slowed responses and increased attentional lapses during night work

One would expect that generalized cognitive slowing resulting from prolonged wakefulness and circadian misalignment would slow response times in perceptual tasks during night work. We did observe substantial impairment in the PVT RT and lapses during night work [23], [24], [45], [46]. Most notably, the nocturnal increase in lapses was most pronounced on the first night shift showing a threefold increase from daytime baseline (Figure 3 right panel). However, as Dinges has long argued [47], [48], lapses capture only part of the story and the extent of cognitive slowing during night work is best seen in changes in the RT distribution. We observed a shift to the right in the entire RT distribution during the night work (Figure 3 left panel). Even the fastest response times were significantly slower during the night shifts. It is noteworthy that the ability to detect a stimulus with an abrupt onset (such as in the PVT) is considerably compromised during night work and this may be even more pronounced during extended wakefulness such as the transition from day to night shift. This slowing of response times is undeniably a hazard, particularly in service-oriented jobs with round-the-clock operations like medicine, transportation, power plant monitoring, and law enforcement where peak functioning at all times is critical.

### Is the first night shift always vulnerable to acute cognitive impairment?

We have shown here that the combination of circadian misalignment and acute sleep deprivation can leave individuals vulnerable to response slowing, speed–error trade-off (fast but sloppy search) in visual selective attention and lapses of attention. Some of these deficits are more serious on the first night of work. While these results together with data from other studies [24], [41] suggest that the risk for errors and accidents may be substantially higher on the first night shift than on any other night shift, there is some evidence to the contrary from field studies [44].

A meta-analysis of data from several studies by Folkard et al [44] indicated that the risk for accidents and injuries can increase over successive shifts, with the increase being greatest during night shifts. Our study was specifically designed to examine the cost of rotating onto the night shift directly from the day shift. In contrast, in most field studies workers start their night shift after a weekend, or after a day or an afternoon shift and the Folkard et al [44] analysis did not separate the data based on when the rotation to a night shift occurred. This constrains any interpretation about the risks of transitioning into night shifts following a day shift from their analysis, because the amount of wakefulness prior to starting night work was variable and not controlled. Moreover, because workers in field setting have an irregular or variable sleep schedule they are likely to suffer from circadian misalignment and increasing sleep loss over the course of several night shifts. Such chronic partial sleep loss can lead to deteriorating cognitive functioning, which is then exacerbated by performing at night because the magnitude of the circadian variation on cognitive performance becomes more pronounced with increased sleep pressure. Under such circumstances individuals could experience an increased risk for accidents and injuries over several shifts [44]. In contrast, in our study we controlled the duration and timing of scheduled rest for every day and night shift, and this likely allowed our subjects greater amounts of sleep and a greater circadian adjustment than is experienced by workers in uncontrolled field studies. These findings argue for adhering to a fixed sleep schedule on night shifts to mitigate the nocturnal cognitive decline seen in field studies of shift work.

### Reducing performance impairment during night work

It is clear that working at night poses a significant hazard to the safety and efficiency of workers as demonstrated by both field and laboratory studies [23], [24], [41], [49]–[55]. Because performance in many jobs where night work is prevalent involves some form of visual search (e.g. medical care, airport baggage screening, law enforcement, power plant monitoring and air traffic control), our results seem to have important practical relevance. Countermeasures that optimize appropriate circadian entrainment and minimize homeostatic sleep drive on night shifts (e.g. bright light exposure, fixed sleep schedule, exercise, exogenous melatonin and/or naps) have been shown to facilitate the transition to night work [1], [2], [24], [49], [56]–[58]. However, because appropriate circadian realignment with reduced homeostatic sleep drive is difficult to achieve in a single night, further research evaluating the effectiveness of additional countermeasures such as naps prior to the start of the first night shift [59], or wake promoting therapeutics is required [60], [61]. Over the course of successive night shifts countermeasures such as scheduled sleep may only partially improve cognitive functioning. For example, in our study despite an 8 hour scheduled sleep episode [34], loss of sensitivity in the search task and attentional lapses on the PVT on the third night shift were significantly greater than daytime baseline levels (Figures 2 and 3) even in subjects who were scheduled to sleep in later in the afternoon. It is likely that a combination of two or more countermeasures may be more effective than a single countermeasure, although additional studies are needed to test this.

Consistent with previous findings we found substantial impairment in the speed and accuracy of response to stimuli, coupled with a decrease in subjective alertness during night shifts [23], [24], [45], [46]. However, the fact that some of these deficits were most pronounced on the first night shift warrants further investigation of cognitive impairment during this shift. It would be informative to concentrate such efforts on understanding nighttime deficits in specific cognitive processes, rather than merely documenting global performance impairment. As it is clear that standard countermeasures which facilitate adaptation to night work may not be effective on the first night shift, it will also be important to develop countermeasures targeted to the specific cognitive deficits on this transition shift. Some future direction in this regard could involve controlled studies with various shift rotations, different sleep schedules and shift timings.

## Methods

### Participants

Eleven men and seven women (26.1±4.8 years) participated in the study. All were healthy and free from medical and psychiatric disorders as determined from a screening evaluation, which included a complete physical examination, clinical biomedical tests on blood and urine, an electrocardiogram, and psychological tests (MMPI and Beck Depression Inventory). Each subject gave written informed consent before starting the study protocol which was approved by the Human Research Committee of the Partners HealthCare System, and was in accordance with the Declaration of Helsinki.

### Procedures

The protocol was a rotating shift work simulation, consisting of four consecutive days of work in the laboratory immediately followed by three consecutive nights of work in the laboratory, and a 38-hour constant routine at the end of the protocol. This is described in greater detail in Santhi et al. [34]. Each subject was assigned to his or her own light-and sound-proof study room for the laboratory portions of the study. Day work was scheduled from 07:00-15:00 and night work from 23:00–07:00. Average illumination in the room during work shifts was typical for indoor lighting (77.03±33.43 Lux [25 µW/cm^2^] measured at a height of 137 cm in a horizontal direction) and was the same for both day and night shifts. Each work episode consisted of four two-hour sessions of a 90-minute battery of computerized cognitive tasks, followed by a 30-minute break. This battery included two selective attention tasks, conjunction search (Figure 1 top, left panel) and spatial configuration search (Figure 1, top right panel), the Psychomotor Vigilance Task, and a visual analog scale for measuring subjective alertness.

The scheduled sleep episodes lasted eight hours. During day work, all subjects were required to remain in bed in the dark from 22:00–06:00 and began the work shift an hour later. On the fifth day subjects woke up at 0600 and started their first night shift seventeen hours later at 2300. Thus, the first night shift in the protocol started 32 hours after the end of the fourth day shift with an intervening 8 hours sleep episode at habitual bedtime. Following the first night shift, subjects were assigned to one of two fixed sleep schedules: Evening Sleep (1400–2200) or Morning Sleep (0800–1600) [34]. Subjects were in the laboratory during work and sleep episodes. Between the work and sleep episodes, subjects left the laboratory and were free to engage in normal activity provided they did not nap. We ascertained compliance by continuously monitoring activity with a wrist actigraph, also equipped with a light sensor (Actiwatch-L, MiniMitter, Sun River, OR).

### Cognitive Task Battery

Visual Search Tasks: Both search tasks required subjects to report on the presence or absence of a target object in an array of simultaneously presented distractors. There were two search tasks, administered in separate blocks. In the conjunction search task (Figure 1, top left panel), the target was a red vertical bar measuring 0.4°×2.1° of visual angle and the distractors were equal numbers of green vertical and red horizontal bars measuring 2.1°×0.4° of visual angle. In the spatial configuration search task (Figure 1 top, right panel), the target was a white block numeral ‘5’ and the distractors were white block numeral ‘2s’, both measuring 0.8°×1.2° of visual angle. In both tasks the stimuli were presented on a black background. A single target was present on 50% of the trials; the remaining trials contained only distractors. The set size was varied from 10 to 40 items in increments of 10. Stimulus locations were determined randomly on each trial. Each trial was preceded by a white fixation plus sign for 500 ms. Subjects were required to indicate whether or not the target was present at the end of the trial by pressing the ‘quote ‘ key for ‘yes’ and the ‘a’ key for ‘no’ responses. There were 100 trials per session and subjects were instructed to respond as quickly and as accurately as possible. *Feedback* was provided at the end of every trial in the form of *RT and accuracy of response*.

We measured RT, slope and intercept of the RT x set size function (computed by regressing RT on set size), and d′ (the signal detection sensitivity parameter computed by transforming accuracy scores [36]). The value of d′ is independent of response bias (the tendency to favor either ‘yes’ or ‘no’ responses), and, unlike raw accuracy, is normally distributed.

Psychomotor Vigilance Task (PVT): This vigilance task required subjects to monitor the center of the display for the appearance of a stimulus, which in our study was a stream of digits which began counting upwards, indicating elapsed time in milliseconds (ms) since signal onset. The subjects were instructed to press a button as soon as the stimulus was detected, at which point the digit stream stopped giving subjects instant reaction time (RT) feedback. The inter-stimulus interval varied randomly between 1000 ms and 9000 ms, and the task lasted twenty minutes. Measures of vigilance included the RT percentile distribution and frequency of lapses (threshold for a lapse: 90th percentile of baseline RT).

Subjective Alertness Scale: We also administered a visual analog scale at the start of each cognitive battery session. The scale allowed us to assess seventeen dimensions of subjective mood. For each dimension, a 100 mm line labeled at either end with the extremes of a subjective dimension (e.g. ‘alert’-‘sleepy’; ‘happy’-‘sad′ etc) was presented on the computer monitor. Subjects used a mouse to move a cursor with a mouse to the point on the line that represented their current state along the dimension. Here we report data from the ‘alert'-‘sleepy’ dimension.

### Supplementary Information

The following suitably anonymized data sets (listed below), on which the analyses reported here were done, will be made available to interested members of the scientific community (as per PLoS policy) as files in a pdf format, upon email request to the following address: nsanthirics.bwh.harvard.edu.

1. Conjunction Search RT.pdf
2. Conjunction Search slope-intercept.pdf
3. Conjunction Search dprime.pdf
4. Spatial Configuration Search RT.pdf
5. Spatial Configuration slope-intercept.pdf
6. Spatial Configuration dprime.pdf
7. PVT lapses.pdf
8. PVT RT.pdf
9. Alertness.pdf
